# Using supervised learning to select audit targets in performance-based financing in health: An example from Zambia

**DOI:** 10.1371/journal.pone.0211262

**Published:** 2019-01-29

**Authors:** Dhruv Grover, Sebastian Bauhoff, Jed Friedman

**Affiliations:** 1 Kavli Institute for Brain and Mind, University of California, San Diego, CA, United States of America; 2 Harvard T.H. Chan School of Public Health, Boston, MA, United States of America; 3 Development Research Group, World Bank, Washington, DC, United States of America; Clemson University, UNITED STATES

## Abstract

Independent verification is a critical component of performance-based financing (PBF) in health care, in which facilities are offered incentives to increase the volume of specific services but the same incentives may lead them to over-report. We examine alternative strategies for targeted sampling of health clinics for independent verification. Specifically, we empirically compare several methods of random sampling and predictive modeling on data from a Zambian PBF pilot that contains reported and verified performance for quantity indicators of 140 clinics. Our results indicate that machine learning methods, particularly Random Forest, outperform other approaches and can increase the cost-effectiveness of verification activities.

## Introduction

Performance-based financing (PBF) is a contracting mechanism that aims to increase the performance and quality of service providers. PBF programs for health care services in low and middle-income countries typically offer financial incentives to health care facilities for the provision of a defined set of services, with an adjustment to the bonus payment based on a broad measure of quality [1]. For example, a PBF program may offer a bonus payment of $5 for each delivery in a clinic, and scale down the bonus if the clinics’ quality is found to be low. In recent years, PBF has generated substantial interest among policy-makers in low- and middle-income countries. Donors and international organizations are actively engaged in supporting countries in developing, implementing and evaluating PBF programs; for instance, as of 2015 a dedicated trust fund at the World Bank alone supported 36 PBF programs [2].

Regular verification by an independent third-party is a central component of PBF, as of all contracts that condition compensation on performance and must contend with asymmetric information between the provider and payer. Providers may react to the incentives not only by increasing performance but also by gaming, e.g., by deliberately over-reporting relative to their actual performance. Verification serves to establish the veracity of performance data (the basis of payment) and to mitigate incentives for gaming by introducing a threat of detection with consequent penalties for identified over-reporting. Verification also serves to help under-reporting of providers (e.g., through misunderstandings or data entry errors) and promote a focus on results.

Effectively targeting verification activities is an important concern for PBF programs. In order to balance the costs and benefits of verification activities, these programs need approaches that detect and deter misreporting by focusing on a sample of providers [1]. However, sampling schemes vary in their performance. For instance, simple random sampling is only effective in identifying misreporting if either the sample or the proportion misreporting are large, as otherwise the odds of capturing those facilities are very small. Sampling performance can be increased by incorporating background and contextual knowledge and using that information to produce models that encode the relationships in the data. This latter sampling problem is a natural application of machine learning, as these techniques use automated learning to identify data attributes that are empirically relevant based on past observations and can achieve highly accurate predictions. This, in turn, can tailor verification activities to high-risk facilities and thus lower cost and/or increase the precision of verification.

Here, we compare strategies for targeted sampling of health clinics for third-party verification of PBF quantity indicators, with a view toward increasing the cost-effectiveness of these essential program activities. Specifically, we compare the performance of a random sampling-based approach (with and without stratification) with four common supervised-learning based classification methods in correctly identifying health clinics that over-reported service volumes relative to verified data: Naïve Bayes, Logistic Regression, Support Vector Machines and Random Forest. We apply these methods to reported and verified data from a PBF program in Zambia and evaluate each method on a range of performance measures. Our results indicate that machine learning methods, particularly Random Forest, outperform other approaches and, as result, can substantially increase the cost-effectiveness of verification activities.

## Background

### Machine learning applications to health systems in low and middle-income countries

Although many high-income countries have started to apply machine learning to different parts of their health systems, including health insurance claims [3], there are currently few examples of such applications in low and middle-income settings [4]. There are several reasons for the low uptake, including different needs, a lack of relevant data and implementation challenges. Many countries only now shift from insurance systems that are input-financed to systems that reimburse health care providers based on specific activities they performed and report. Although these health systems have a policy need to examine the veracity of reported claims, they tend to have low capacity and weak data systems that are often not designed to support audits and verification [5,6]. Instead, these health systems identify suspicious records using trigger rules, such as multiple admissions for the same patient for the same procedure or whether a hospital’s reported patient volume exceeds its capacity [7].

### Verification in PBF programs

PBF offers financial incentives for increasing the quantity and/or quality of a specific set of health care services. Rewards for increasing quantities are generally “fee for service” payments, so that providers receive a bonus for every additional service they report. Many programs inflate or deflate the quantity bonus based on a broad index of quality.

Verification serves to counteract the incentive to over-report and, in some cases, to fulfill the fiduciary responsibilities of the implementing agency. A typical PBF program has three layers of verification [1,8,9]. First, district or provincial supervisors visit all facilities on a monthly or quarterly basis to confirm the accuracy of the reported quantity data. Second, district teams visit all facilities on a quarterly basis to complete a quality assessment. Third, an independent third-party such as a community or non-governmental organization conducts quarterly counter-verification visits to a (often random) sample of facilities. Core counter-verification activities include reconciling health service records at different points in the reporting chain, from individual services recorded in patient attendance books up to service aggregation sheets at the district level. Additional activities can include client tracer surveys that re-contact patients to verify the receipt of recorded service and inquire about patient satisfaction. Discrepancies between the reported and verified data result in penalties, such as deductions from the PBF bonus or exclusion from future rounds of the program.

PBF programs vary in their approach to targeting facilities for counter-verification and the associated costs. In Burundi, the third-party agency randomly selects one district in each of four provinces every quarter; the sample of provinces is randomized but rotating so that all provinces are visited within a year [10]. The district hospital and a random sample of 25% of health centers are audited in the selected districts using quantity verification, technical quality assessment and household surveys. Facilities with positive or negative discrepancies of more than 5 percent should be subject to fines. The penalty increases in the size of the discrepancy so that, e.g., 10 percent of the PBF bonus is withheld if the discrepancy is 10–20 percent. In Benin, community-based organizations are contracted to conduct quarterly counter-verification through unannounced visits to facilities as well as tracing a random sample of patients to confirm the receipt and experience of service [9]. For a series of detailed case studies of verification in PBF, see [11].

Counter-verification can represent a substantial share of overall program financial costs as well as staff time and effort, so that improved targeting could yield important savings. Although there is no systematic overview of verification costs in PBF, data from individual programs suggests that the costs vary substantially with the program design. In Burundi in 2011, the financial costs of these activities represent 1 percent of overall spending, not accounting for the time costs of district and facility staff [10]. In Benin in 2013–2014, the financial costs represent about 30 cents for every dollar paid to providers in bonuses, and there are additional time costs of district and facility staff [9]. In Argentina’s Plan Nacer, which incentivizes provinces rather than facilities, verification costs may be equivalent to about 10 percent of the maximum bonus payments.

### Zambia’s performance-based pilot

Zambia operated a PBF pilot project from 2012 to 2014 in an attempt to realign health financing towards outputs rather than inputs, and to address various health system concerns such as relatively low coverage of key maternal and child health services. The pilot operated in public health centers in 10 rural districts, covering a population of 1.5 million, or about 11% of Zambia’s population [12]. It comprised two core features, financial rewards and equipment upgrades. Specifically, the program offered varying fee-for-service bonus payments for indicators measuring the quantity of nine maternal and child health (see S1 Table) and 10 quality domains covering aspects of both structural and process quality. Health centers also received emergency obstetric care equipment. In addition, participating health centers were subject to enhanced monitoring.

The financial rewards from the PBF were substantial, with an individual staff’s bonus representing on average 10% of government salary [12]. An evaluation of the pilot based on independent population surveys found gains in selected targeted indicators, such as the rate of facility deliveries [13]. Other targeted indicators, especially those at already high levels of coverage such as ante-natal care, saw little change.

The program extensively audited reported data through continuous internal verification and a one-off external process. Dedicated district steering committees served as internal verifiers, reconciling the facility reported information which served as the basis for incentive payments with the paper based evidence of services provided at the facility. An independent third party verified reports by primary health clinics on a sample basis in a one-off exercise after two years of program operation; there were no other audit visits. The total cost of this one-off external verification came to 1.5% of the total US$ 15 million spent on the pilot project. The cost of internal verification activities was assuredly greater given the scale of activities involved.

### Conceptual framework

Conceptually, the primary fiduciary objective of counter-verification is to reduce or eliminate over-reporting while minimizing verification costs. The costs of this scheme can be modeled as a simple cost function where the total cost, *tc*, is a linear function of several parameters:

tc=n[p(mc−s)+(1−p)mc]+fc

*n* is the total number of facilities selected into the verification sample. *p* is the proportion of sampled facilities found to have misreported in a given time period (e.g. a quarter). *mc* is the marginal cost of verification at a facility, which is assumed to be the same regardless of whether the facility is a mis-reporter or not. *s* is the financial sanction that the over-reporter must pay to the health system, and *fc* is the fixed cost of verification activities that are constant across time periods.

The financial efficiency gains from improving the accuracy of predicting what facilities are over-reporting (i.e. increasing *p*) while keeping *n* constant arises through two channels: (1) an increase in *p* directly leads to a lower cost of the verification by increasing the amount earned back through sanction payments, (2) an increase in *p* may have a dynamic effect in so far as (a) facilities identified as over-reporters may be less likely to over-report in the future and (b) all facilities, whether included in the sample or not, may be deterred from over-reporting if the likelihood of identification in a future verification is sufficiently high. Accurate prediction should increase this deterrence effect, and future verification efforts can be reduced in size as the incidence of future over-reporting declines.

## Data and methods

### Data from Zambia’s PBF pilot

We use both operational and specially collected data from the Zambia pilot project to evaluate the performance of different classification methods. Although a simulated dataset would have been sufficient for this assessment, the Zambia data are realistic with regards to data attributes and parameters.

Specifically, we combine data from facility reports and a dedicated facility survey, conducted in 2014, that was designed to reproduce the stipulated external verification activities in a sample of facilities. The data collected by the facility survey cover the same indicators and time period as the previously conducted audit exercise, i.e. select services reported in calendar year 2013. The data cover 105 primary health care centers in the 10 PBF pilot districts and 35 centers in another 8 non-pilot districts, for a total of 140 facilities. The population of facilities were stratified by district and then selected on a proportional-to-size basis with respect to the facility catchment area. Reported performance stems from the Health Information Aggregation (HIA) 2 forms in which health facilities summarized services provided for each indicator. Verification data were collected on the complete set of nine incentivized indicators and cover every calendar month of 2013. These data are derived from tally sheets, activity sheets and registers, as these records indicate the individual services delivered to a specific client. These data sources contain the date of the service, client register number, and other information and were used to check errors relating to summing, recording and data entry. The survey team was rigorously trained and had detailed guidelines on how to collect these data.

This study received ethical approval from the Humanities and Social Sciences Research Ethics Committee, University of Zambia (IRB 00006464, IORG: 000376). Written informed consent was collected from all respondents and personal information was kept confidential.

Our focus is on whether or not a facility over-reported. Our primary measure of interest is a binary measure of over-reporting relative to the verified data. We calculate this measure in two steps. First, for each facility in each quarter, we calculate the product of the quantity and price (reward) for each indicator and then sum these products to obtain the total bonus payment based on (separately) the reported and verified data. Second, we calculate the difference in the bonus payments between the reported and verified amounts, and evaluate whether it is substantively large. We classify a facilities’ quarterly report as over-reported if the bonus calculated on the reported data exceeds the bonus calculated on the verified data by 10% or more. The binary indicator and the specific cutoff reflects common practice in operational PBF programs. Our label of “over-reporting” emphasizes that a regulator would primarily be concerned about cases when a facility’s report exceed the actual volume of services delivered, because of the associated over-payment. The cutoff of 10% allows for possible leniency for smaller mismatches and generates sufficient variation in the data.

Tables 1 and 2 illustrate the structure of the Zambia data over the four quarters of 2013. 15–23 percent of facilities over-report in a given quarter. There is a strong correlation of over-reporting over time: of the facilities that over-report in quarter 1, 58 percent also over-report in quarter 2, and 42 percent also over-report in quarters 3 and 4. Table 2 shows that about 58 percent of facilities never over-report and only 4 percent over-report in all four quarters.

**Table 1:** Overview of data from Zambia pilot.

	Quarter			
** **	**1**	**2**	**3**	**4**
Percent over-reporting	18.6	15	22.9	20
Count	140	140	140	140
**Percent of facilities over-reporting if also over-reporting in…**				
Quarter 1	100	57.7	42.3	42.3
Quarter 2	71.4	100	66.7	47.6
Quarter 3	34.4	43.8	100	43.8
Quarter 4	39.3	35.7	50	100

**Table 2:** Distribution of facilities that over-report.

	N	Percent
Never	81	57.9
One quarter	32	22.9
Two quarters	12	8.6
Three quarters	9	6.4
All four quarters	6	4.3

### Classification methods

We evaluate the performance of sampling-based approaches in classifying a facilities’ quarterly report as over-reporting, and compare their performance to four alternative machine learning approaches: Naïve Bayes, Logistic Regression, Support Vector Machines and Random Forest. In this section we briefly describe these approaches and considerations for their use.

#### Sampling-based approaches

Random sampling is the current default selection method for counter-verification in many PBF programs [1]. This approach is routinely recommended for auditors in related settings, including fraud detection in insurance programs and financial audits (see, e.g., [14]).

In this study, we examine how well four approaches to random sampling perform with regards to identifying clinics that over-report. First, we use simple random sampling to determine the probability of an over-reported event, wherein 50% of the clinics are chosen repeatedly at random. Second, we stratify the sample by district and then use simple random sampling to select 50% of clinics within these strata. This approach ensures that counter-verification takes place in all districts. Third, we leverage knowledge of what clinics previously over-reported. Specifically, we use simple-random sampling to draw half the audit sample from those clinics that over-reported in the immediate prior quarter, and simple random sampling to draw from the remaining clinics. The overall sample size is 20% of all clinics, i.e., 28 clinics. Fourth, we select up to 28 clinics that were prior offenders. If the number of prior offenders is greater than 28, we use simple-random sampling to select the target number. If the number is less than 28, we randomly sampled from the remaining facilities to achieve the target number.

We report the accuracy of the sampling-based approaches as averages of 1000 independent sampling iterations without replacement.

#### Supervised machine learning approaches

Supervised learning is a class of machine learning algorithms that use labeled examples to infer a relationship between input and output variables, and then use that inferred relationship to classify new examples. The underlying basis of these algorithms is to generalize from training data to prediction of class labels of unseen instances from a test set.

In the context of verification in PBF, these training data are a subset of facility-specific data points (input) that contain a binary indicator for whether or not a facility over-reported (output). The algorithm learns from these data which facilities are at risk of over-reporting, and applies this learning to predict this risk for other facilities not included in the training data.

For the below analyses, we used as input features the reported and verified values for the nine quantity measures that were rewarded in the RBF program, along with the district identifier and a categorical variable indicating the treatment arm from a related audit experiment. The audit experiment randomly varied the probability of audit (10, 30 or 100 percent) in the RBF areas while clinics in non-RBF areas had a zero percent probability of audit. Facilities were told what their specific audit probability was but all PBF facilities received a visit by external auditors. In a supplemental analysis, we expanded this set of 22 covariates to include an additional 6 facility-level covariates (see below).

We chose four traditional supervised machine learning approaches, each with unique strengths and weaknesses, to test their efficacy for our classification problem. First, Naïve Bayes, a simple and efficient classification technique that involves the application of Bayes theorem (with a strong independence assumption between features), wherein the probability of a feature is determined using prior knowledge of conditions that might be related to that feature [15,16]. Second, Logistic Regression, which uses a logistic function at its core to estimate a relation between the binary classification and its possible predictors [17,18]. Third, Support Vector Machine (SVM), a discriminative non-probabilistic classifier formally defined by a hyperplane that maximizes the separation between the two classes [19]. Fourth, Random Forest, an ensemble-based classification algorithms that use multiple voting mechanisms for decision trees to improve their accuracy and reduce variance [20,21].

## Analysis

We assess the performance of the four classifiers used in this study across the following five performance metrics: prediction accuracy, recall, precision, F1-score and area under the receiver-operating-characteristic (ROC) curve (AUC) [22]. If the classifier is required to perform equally well on the positive class as well as the negative class, ROC curves that plot the true positive rate (TPR) against the false positive rate (FPR) serve well for visualization and evaluation. However, if there is greater interest in the model’s performance on the negative class, to make sure every positive prediction is correct (precision), and that we get as many positive class predictions correct as possible (recall), a precision-recall curve is better suited. F1-score is an indicator to measure the accuracy of a dichotomous model and considered as the harmonic average of model precision and recall. Algebraically:

$$accuracy=\frac{correctly\;classified\;samples}{total\;number\;of\;samples}$$

$${precision}_{a}=\frac{samples\;correctly\;classified\;as\;class\;a}{samples\;classified\;as\;class\;a}$$

$${recall}_{a}=\frac{samples\;correctly\;classified\;as\;class\;a}{samples\;of\;class\;a}$$

$$F1-score=2\sum \frac{{recall}_{a}\times {precision}_{a}}{{recall}_{a}+precision_{a}}$$

We used 10-fold cross-validation on the Q1 dataset (140 cases), in which the original sample is randomly partitioned into 10 equal size subsamples. Of the 10 subsamples, a single subsample is retained as the validation data for testing the model, and the remaining 9 subsamples are used as training data. The cross-validation process is then repeated 10 times, with each of the 10 subsamples used exactly once as the validation data. We then averaged the 10 results to produce a single estimation. Given that the size of our training dataset is relatively small, the advantage of this method is that all observations are used for both training and validation, and each observation is used for validation exactly once. Similarly, we report the accuracy of the sampling-based approaches as the averages of 1,000 independent sampling iterations.

For each metric we found the best parameter settings for each algorithm using the validation data set aside by cross-validation, then report that model’s normalized score on the test set. Following training, we then used the estimated models to further classify data from Q2-Q4, i.e., we assess the models’ prediction accuracy in subsequent periods. All statistical analyses were executed in Matlab R2017b [23].

## Results

**Fig 1:**
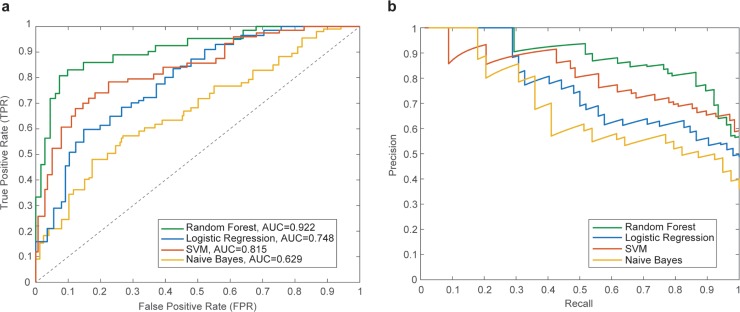
(a) Average ROC curves and their corresponding AUC values for random forest, logistic regression, support vector machine and naïve Bayes classifiers using cross-validation with training data. (b) Precision-Recall curves.

Fig 1A shows the ROC curves and their corresponding AUC values for random forest, SVM, logistic regression and Naïve Bayes classifiers. Fig 1B shows the corresponding Precision-Recall curves for the same classifiers.

Table 3 shows the normalized scores for each supervised machine learning classifier across five performance metrics. Each entry in the table averages these scores across the ten trials. In general, Random Forest performs better than other approaches, achieving an additional ~22% accuracy beyond other classifiers under consideration. This classifier also receives higher F1-score and AUC than other algorithms.

**Table 3:** Average performance metrics of the four machine learning classifiers. The best metrics in each column are shown in bold.

Model	Accuracy	Precision	Recall	F1-score	AUC
Logistic Regression	0.584	0.627	0.713	0.509	0.748
Naïve Bayes	0.552	0.523	0.628	0.425	0.629
SVM	0.647	0.691	**0.874**	0.651	0.815
Random Forest	**0.866**	**0.896**	0.851	**0.821**	**0.922**

Table 4 shows the prediction accuracy of the four supervised learning algorithms and the four sampling approaches. The classifiers were trained on quarter-1 data, and then used to further predict classification of data from quarters 2–4. As in Table 3, Random Forest performs best with almost 87% prediction accuracy for the quarter-1 data and 77–89% accuracy for data from quarters 2–4. SVM also performs well relative to other methods but has substantively lower performance than Random Forest at 64% in the cross-section and 49–58% in the time-series. The sampling methods perform worse than most of the supervised learning approaches (except logistic regression): Simple random sampling (SRS) and SRS with district stratification have a low predictive accuracy. Sampling using historical information about over-reporting performs better, reflecting the correlation of over-reporting over time shown in Table 1. Revisiting the top-offenders further boosts the prediction accuracy. Note that Table 1 shows the accuracy of revisiting all offenders of a previous quarter, without sampling. Of those facilities that over-reported in the first quarter, 57.7%, 42.3% and 42.3% also over-report in quarters 2–4, respectively.

**Table 4:** Prediction accuracy performance of different approaches.

Approach	Prediction of over-reported event			
	Q1	Q2	Q3	Q4
**Sampling approaches**				
SRS	18.77%	14.98%	22.56%	20.04%
SRS with district stratification	18.83%	15.21%	23.22%	19.9%
SRS of offenders & non-offenders	-	34.5%	36.5%	27.87%
SRS of only offenders	-	44.5%	42.19%	38.81%
**Supervised learning**				
Logistic Regression	58.42%	32.84%	31.28%	34.76%
Naïve Bayes	55.24%	46.15%	32.05%	41.3%
SVM	64.75%	58.02%	49%	52.26%
Random Forest	86.6%	89.18%	84.92%	77.31%
Random Forest with district	87.84%	86.19%	81.99%	76.96%
Random Forest with intervention	85.08%	82.29%	77.83%	73.08%

Note: Accuracy is calculated as average of 1,000 independent sampling without replacement iterations for SRS, and 10-fold cross-validation for supervised learning.

For each of the supervised learning classifiers, we thoroughly explored the parameter space related to the classification methods. For instance, we performed Naïve Bayes modeling as single normal, with kernel estimation, or discretizing them with supervised discretization. In Logistic Regression, we tested both un-regularized and regularized models, varying the ridge parameter by a factor of 10 from 10^-5 to 10^-2. For SVMs, we used the following kernels: linear, polynomial degree 2 and 3, radial with width ranging from 10^-3 to 1 by a factor of 10. For Random Forest, we used the Breiman-Cutler version with btrees of sizes (50, 100, 200, 500) and size of feature set considered at each split from 1–9. One-hot encoding was used to binarize categorical variables for treatment of feature importance bias with Random Forests. S1 Fig shows the tuning of the Random Forest *mtry* parameter (number of variables randomly sampled at each split) for a range of *ntree* (number of trees to grow). The most accurate values for *ntree* and *mtry* were 200 and 3, respectively, with an accuracy of 84.4%.

We also examined whether additional covariates would improve the predictive accuracy of the supervised learning algorithms. S2 Table shows the predictive accuracy when also accounting for the facility type, managing authority, location, size of the catchment population, and number of established and filled staff posts. Incorporating these covariates slightly reduced the performance for all algorithms. S2 Fig shows the importance of covariates in the Random Forest algorithm and indicate that these additional covariates contribute little to the predictive accuracy.

These results suggest that using supervised learning instead of random sampling can substantially lower the costs of verification. As the total cost formula suggests, the actual savings depend on several parameters that will vary by context. To illustrate the potential savings, we consider the costs to of verification at one facility (n = 1) for the first quarter, using the average costs instead of total costs of verification. Using reasonable estimates of the average cost of verification ($1,600) and the marginal cost ($800 per facility), and that over-reporting facilities lose their entire quarterly bonus (about $1,900 in the first quarter), changing from SRS (detection probability p = 19%, see Table 4) to Random Forest (p = 88%) would reduce the costs of verification per facility by about two-thirds (from $2,039 to $728).

## Discussion

Verification of reported performance is a crucial issue in PBF in health, given that the payment incentives may not only encourage increased performance but also over-reporting. Independent verification serves to deter over-reporting and to ensure that payments reflect actual performance.

We described several approaches to identify the set of health clinics that should be audited, and tested performance of these approaches on self-reported and verified data for 140 clinics in Zambia. Our results indicate that sampling-based approaches do not perform well even with large sample sizes. Algorithm-based supervised learning methods perform substantially better, especially Random Forest which–in our data–has a prediction accuracy that remains high even over time. The finding that Random Forest outperforms a regression approach such as Logistic Regression indicates that over-reporting is a highly non-linear function of covariates–information commonly observed in administrative data or facility surveys–and consequently predictions from traditional regression analysis will not be particularly accurate.

These high-performing methods are feasible in operational PBF settings: they use existing data, and can be updated as new information becomes available. Indeed, unlike sampling-based approaches, the supervised learning methods are likely to further improve as new and additional data becomes available. These methods can also be made user friendly and automated, by drawing data from existing data systems, such as the electronic District Health Information System (DHIS-2) or dedicated PBF data portals, and outputting the list of facilities to be visited by verification teams.

Improving the prediction accuracy yields several benefits to PBF programs. It leads to potentially substantial cost savings as, on the margin, each detected case will result in a penalty that helps defray the costs of the verification activities. Methods with high accuracy may also be more effective at deterring over-reporting over time. They will also reduce the time that staff of correctly reporting clinics spend (unnecessarily) to support verification activities. Finally, better detection may also be perceived as more fair and improve the acceptance of PBF among the clinics and policy-makers.

There are several directions for future research. First, the various approaches could be tested on different data, either from other real-life PBF program or simulated data, and with different definitions of what constitutes “over-reporting”. Our findings are applicable to a particular setting and program design, and the performance of the methods may vary across contexts. Second, there may be other approaches that could perform well but that we did not test in this study. For instance, sampling-based approaches could use more productive strata than districts, e.g., strata constructed using machine learning or principal-component analysis of covariates that are predictive of over-reporting. Third, future testing or implementations could use additional covariates to improve the accuracy of the supervised learning methods, e.g., district and facility characteristics such as size, staffing and remoteness, or contextual information such as area socio-economic factors. Some of these data are readily available in settings with electronic health management information systems. As our example shows, adding covariates is not always an improvement and, indeed, can worsen predictive accuracy. Finally, additional research may be required on the behavioral response of clinics to the performance of the verification scheme and how the scheme could be adapted over time to address these responses as well as general improvements in reporting accuracy. We relied on quarter-1 data to predict over-reporting in subsequent quarters, so that any response after quarter 1 would not impact the predictions of the algorithm-based models. However, behavioral responses could affect the data basis for models built on later data; the sampling-based models are immune to this concern. For this reason, a hybrid approach may involve periodic retraining of the learning algorithm on a new random sample drawn from the population of participating clinics.

## Conclusion

Overall our findings suggest that supervised learning approaches, such as Random Forest, could substantially improve the prediction accuracy of counter-verification in PBF and thus increase the cost-effectiveness of verification. These methods are operationally feasible, especially in settings with electronic routine reporting systems.

## Supporting information

S1 Fig Tuning of random forest *mtry* parameter (number of variables randomly sampled at each split) for a range of *ntree* (number of trees to grow). (EPS)

S2 Fig Feature importance criteria for random forest classification with basic set of variables. Note: “ts” refers to tally sheet; hia refers to Health Information Aggregation 2 forms.
(EPS)

S3 Fig Feature importance criteria for random forest classification with expanded set of variables. Note: “ts” refers to tally sheet; hia refers to Health Information Aggregation 2 forms.
Q6 = type of facility (7 levels plus “other”, from central hospital to rural health post).
Q7 = managing authority (government, mission/FBO, private, military, other).
Q8 = location (rural, peri-urban, urban).
Q9 = size of catchment population.
est = total number of established staff posts.
fill = total number of filled staff posts.
(EPS)

S1 Table Rewarded indicators in Zambia’s 2012–2014 pilot PBF. * Approximate value. Source: [13].
(DOCX)

S2 Table Prediction accuracy performance of different approaches with expanded set of variables. (DOCX)
