# Supplementary material for: Using supervised learning to select audit targets in performance-based financing in health: An example from Zambia
Source: PLoS One. 2019 Jan 29;14(1):e0211262. doi: 10.1371/journal.pone.0211262 (PMC6350980; doi:10.1371/journal.pone.0211262)
Supplement: S1 Table — * Approximate value. Source: [13]. (DOCX) [file pone.0211262.s004.docx]

S1 Table. Rewarded indicators in Zambia’s 2012-2014 pilot PBF

| # | Indicator | Fee (Kwacha) | Fee (USD*) |
| --- | --- | --- | --- |
| 1 | Curative Consultation | 1,000 | 0.20 |
| 2 | Institutional Deliveries by Skilled Birth Attendant | 32,000 | 6.40 |
| 3 | ANC prenatal and follow up visits | 8,000 | 1.60 |
| 4 | Postnatal visit | 16,500 | 3.30 |
| 5 | Full immunization of children under 1 | 11,500 | 2.30 |
| 6 | Pregnant women receiving 3 doses of malaria IPT | 8,000 | 1.60 |
| 7 | FP users of modern methods at the end of the month | 3,000 | 0.60 |
| 8 | Pregnant women counselled and tested for HIV | 9,000 | 1.80 |
| 9 | HIV Exposed Babies administered with Niverapine and AZT | 10,000 | 2.00 |

* Approximate value. Source: [13].
