# Supplementary material for: Using supervised learning to select audit targets in performance-based financing in health: An example from Zambia
Source: PLoS One. 2019 Jan 29;14(1):e0211262. doi: 10.1371/journal.pone.0211262 (PMC6350980; doi:10.1371/journal.pone.0211262)
Supplement: S2 Table — (DOCX) [file pone.0211262.s005.docx]

S2 Table. Prediction accuracy performance of different approaches with expanded set of variables

| Approach | Prediction of over-reported event | | | |
| --- | --- | --- | --- | --- |
|  | Q1 | Q2 | Q3 | Q4 |
| Logistic Regression | 49.31% | 28.64% | 26.49% | 29.14% |
| Naïve Bayes | 49.97% | 40.58% | 33.16% | 38.68% |
| SVM | 58.38% | 52.97% | 45.74% | 49.82% |
| Random Forest | 82.72% | 79.38% | 74.26% | 71.16% |
